# Reply to the letter for the manuscript “The effect of proprioceptive vestibular rehabilitation on sensory-motor symptoms and quality of life”

**DOI:** 10.1055/s-0046-1817807

**Published:** 2026-04-29

**Authors:** Gülfem Ezgi Özaltın, Burcu Talu, Tuba Bayındır

**Affiliations:** 1 Inonu University, Faculty of Health Sciences, Physiotherapy and Rehabilitation Department, Malatya, Turkey.; 2 Yüksek İhtisas Unıversity, Medicalpark Ankara Hospital, Ear Nose and Throat Clinic, Ankara, Turkey.

Dear Editor,

We would like to thank Partiksha and Sadhu for their interest in our article titled “The effect of proprioceptive vestibular rehabilitation on sensory-motor symptoms and quality of life”. Their comments give us the opportunity to discuss this article further. You can find the answers to the current questions below.

- Before the study started, a power analysis was performed using α = 0.05 and 1-β (power) = 0.80, assuming that the change in Dizziness Handicap Inventory (DHI) score was 27 on average. According to the power analysis, it was calculated that a total of at least 27 individuals should be included, with at least 9 individuals in each group. The minimum sample size determined at the end of the study was reached. 1 A computerized randomization method (Research Randomizer) was used to assign individuals to groups. This method was preferred to initially ensure balance between the groups and to minimize bias. The numbers assigned to the groups with the Research Randomizer were drawn by the patient in a sealed envelope and assigned to the relevant group. Individuals were included in the study without knowing which group they were in.
- The analysis of our study was conducted by evaluating only individuals who completed the treatment program. In this context, no treatment intention analysis was conducted, which can be considered as a limitation of the study.
- In the current study, after the comparisons between the groups, post hoc analyses were performed to determine which groups the difference was between. The aim was to determine the statistical significance of the results. However, determining the level of clinical effect will contribute to our study. Therefore, Cohen's d effect sizes were calculated and presented in an additional table, the table and the revised version are given in the appendix. These analyses will allow a more comprehensive evaluation of the effect of periferal vestibular rehabilitation.
- In our study, the Adolescent and Adult Sensory Profile was used to evaluate the effects of proprioceptive stimulation on sensory integration. The sensory profile was specified with quantitative outcome measures in the 4-quadrant sensory sensitivity, sensory avoidance, low registration and sensory seeking features. 2 In order to better explain the effect of proprioceptive vestibular rehabilitation on sensory integration, the relevant literature and neurophysiological mechanisms are discussed in the discussion section. In our study, it was aimed to obtain plasticity in the vestibulo-autonomic reflex and VSR and vestibulo-collic reflex after vestibular rehabilitation. The improvement in the untreated group can be explained by the central compensation mechanism. The better results in the treatment groups can be explained by the strengthening of vestibulo-autonomic and vestibulo-spinal reflex connections and the development of plasticity in vestibular synapses. It was observed that postural and vegetative symptoms disappeared and individuals entered normal/nearly normal sensory patterns according to the sensory profile results. The study emphasizes that sensory problems of individuals with vestibular symptoms should be addressed along with their motor problems. However, more comprehensive studies on this subject in the future will contribute to the literature.
- The intervention period in our study was determined as 8 weeks based on vestibular rehabilitation protocols recommended in the literature. In the literature, it is stated that vestibular rehabilitation programs generally vary between 6-12 weeks and that this period is sufficient to provide clinically significant improvements. 3 In our study, the effects of proprioceptive vestibular rehabilitation were evaluated in the 8-week follow-up period and statistically significant improvements were reported. However, the evaluation of long-term results is an important issue in terms of the sustainability of vestibular rehabilitation. Therefore, the results of our study provide a basis for future studies to investigate long-term effects. We accept that longer follow-up studies are needed in this area and we think that further studies should evaluate the long-term effects of vestibular rehabilitation.
- In our study, it is observed that proprioceptive stimulation added to vestibular exercises positively affects motor skills and balance together with habit adaptation and substitution exercises in individuals with peripheral vestibular hypofunction. The basic mechanism here can be based on neuroplasticity. The vestibular system and the proprioceptive system work together in providing postural control and maintaining balance. Normally, the responses of vestibular neurons are suppressed during movement and balance is maintained through vestibulo spinal reflexes. In this way, it allows the person to make controlled movements towards the target. The vestibular system works integrated with the proprioceptive system and balances the person's own movement and perception of the environment. 4 In animal studies, it has been observed that proprioceptive inputs in the vestibular nuclei increase after peripheral vestibular loss. Stimuli arriving at the vestibular nucleus neurons are transmitted via AMPA receptors that transmit vestibular inputs and NMDA receptors that transmit proprioceptive inputs. Normally, proprioceptive information coming from NMDA receptors is silent. However, after vestibular loss, silent NMDA synapses become active with an increase in the number of AMPA receptors and allow proprioceptive information to reach them. 5 6 7 This process shows that the vestibular system tries to maintain balance by using alternative sensory pathways after the loss. This situation indicates the compensation mechanism and plasticity of the central nervous system. In future studies, more extensive neurophysiological studies are needed in this context. We hope that the findings obtained from the current study will guide new study topics.
